# Radiomics as a Decision Support Tool for Detecting Occult Periapical Lesions on Intraoral Radiographs

**DOI:** 10.3390/jcm15030971

**Published:** 2026-01-25

**Authors:** Barbara Obuchowicz, Joanna Zarzecka, Marzena Jakubowska, Rafał Obuchowicz, Michał Strzelecki, Adam Piórkowski, Joanna Gołda, Karolina Nurzynska, Julia Lasek

**Affiliations:** 1 Department of Conservative Dentistry with Endodontics, Institute of Dentistry, Jagiellonian University Medical College, Montelupich 4, 31-155 Krakow, Poland; barbara.obuchowicz@uj.edu.pl (B.O.); j.zarzecka@uj.edu.pl (J.Z.); mjakubowska@uks.com.pl (M.J.); joanna.golda@uj.edu.pl (J.G.); 2 Department of Diagnostic Imaging, Jagiellonian University Medical College, 30-663 Krakow, Poland; 3 Institute of Electronics, Łódź University of Technology, 93-590 Łódź, Poland; michal.strzelecki@p.lodz.pl; 4 Department of Biocybernetics and Biomedical Engineering, AGH University of Krakow, 30-059 Krakow, Poland; 5 Department of Algorithmics and Software, Silesian University of Technology, 44-100 Gliwice, Poland; karolina.nurzynska@polsl.pl; 6 Faculty of Geology, Geophysics and Environmental Protection, AGH University of Krakow, 30-059 Krakow, Poland

**Keywords:** periapical diseases, apical periodontitis, dental radiography, cone-beam computed tomography, radiomics, artificial intelligence, endodontics

## Abstract

**Background:** Periapical lesions are common consequences of pulp necrosis but may remain undetectable on conventional intraoral radiographs, becoming evident only on cone-beam computed tomography (CBCT). Improving lesion recognition on plain radiographs is therefore of high clinical relevance. **Methods:** This retrospective, single-center study analyzed 56 matched pairs of intraoral periapical radiographs (RVG) and CBCT scans. A total of 109 regions of interest (ROIs) were included, which were classified as CBCT-positive/RVG-negative (onlyCBCT, *n* = 64) or true negative (noLesion, *n* = 45). Radiomic texture features were extracted from circular ROIs on RVG images using PyRadiomics. Feature distributions were compared using Mann–Whitney U tests with false discovery rate correction, and classification was performed using a logistic regression model with nested cross-validation. **Results:** Forty-four radiomic texture features showed statistically significant differences between onlyCBCT and noLesion ROIs, predominantly with small to medium effect sizes. For a 40-pixel ROI radius, the classifier achieved a mean area under the ROC curve of 0.71, mean accuracy of 68%, and mean sensitivity of 73%. Smaller ROIs (20–40 pixels) yielded higher AUCs and substantially better accuracy than larger sampling regions (≥60 pixels). **Conclusions:** Quantifiable radiomic signatures of periapical pathology are present on conventional radiographs even when lesions are visually occult. Radiomics may serve as a complementary decision support tool for identifying CBCT-only periapical lesions in routine clinical imaging.

## 1. Introduction

Periapical bone rarefactions are inflammatory lesions that develop as a consequence of microbial infection of the root canal system, most commonly following pulp necrosis caused by deep caries, trauma, or restorative procedures [1]. Persistent bacterial antigens and their by-products at the apical foramen trigger a host immune response, leading to periapical bone resorption mediated by inflammatory cells and cytokines [2]. Periapical lesions are highly prevalent in adult populations. In a randomly selected cohort, Ödesjö et al. [3] reported that approximately 53% of individuals had at least one root-filled tooth, with periapical radiolucencies present in 31–35% of these teeth. A systematic review and meta-analysis further demonstrated that individuals with diabetes exhibit a 1.4–1.6-fold higher likelihood of presenting periapical radiolucent lesions in root-filled teeth compared with non-diabetic controls [4]. Lesions occur more frequently in molars and premolars, likely reflecting their complex root canal anatomy and higher caries risk, and their distribution is influenced by socioeconomic factors, access to dental care, and geographic variability [5].

Radiographically, periapical bone rarefactions appear as radiolucent areas corresponding to the localized loss of mineralized bone secondary to chronic inflammation [2]. Because these lesions are often clinically asymptomatic, they are commonly detected incidentally during routine radiographic examinations [2]. Conventional periapical radiography remains the first-line imaging modality in endodontic practice due to its wide availability and low radiation dose; however, its diagnostic reliability is inherently limited by the two-dimensional representation of complex three-dimensional structures [6]. Panoramic and intraoral periapical radiographs therefore demonstrate substantially lower sensitivity for detecting periapical lesions compared with cone-beam computed tomography (CBCT), particularly for small lesions and those located in anatomically complex regions [7]. CBCT-based studies have shown that a considerable proportion of periapical lesions remain undetected on conventional radiographs, especially when lesions are confined to cancellous bone or obscured by anatomical superimpositions [8]. These limitations underscore the clinical need for adjunctive, quantitative image analysis approaches capable of enhancing lesion recognition on conventional radiographs, particularly in settings where CBCT cannot be routinely applied [7]. Consistent with these observations, Raghav et al. reported that conventional radiography detects approximately 53% of periapical lesions, thus leaving a substantial proportion of early or subtle lesions undetected [9].

Radiomics is a quantitative image analysis approach that converts standard medical images into many mathematically defined features describing gray-level intensity distributions, texture patterns, and spatial relationships between pixels. By capturing sub-visual image characteristics, radiomics aims to quantify tissue heterogeneity and microstructural alterations that are not discernible through conventional visual assessment [10]. Texture-based radiomic features, including descriptors derived from gray-level co-occurrence, run length, size zone, and dependence matrices, have been widely applied in medical imaging to improve lesion detection and characterization beyond human perception [10]. In dental and maxillofacial imaging, texture analysis has demonstrated diagnostic value for differentiating periapical granulomas from radicular cysts on CBCT images and for assisting in the detection of inflammatory bone changes that are difficult to evaluate visually [11,12]. Previous studies have demonstrated that radiomics applied to cone-beam computed tomography (CBCT) can support several clinically relevant diagnostic and assessment tasks in endodontics and maxillofacial imaging. CBCT-based radiomic texture analysis has been shown to differentiate periapical granulomas from radicular cysts with accuracies exceeding 80% [11], thereby assisting lesion characterization beyond subjective radiologic interpretation. In addition, radiomics has improved the detection and assessment of furcation-related bone defects and inflammatory bone changes that are difficult to evaluate visually on CBCT alone [12]. These applications highlight the potential of CBCT radiomics not only for lesion detection, but also for lesion classification, structural heterogeneity assessment, and decision support in treatment planning and follow-up. Such evidence provides a strong conceptual basis for extending radiomics-based analysis to conventional intraoral radiographs in scenarios where CBCT is not routinely available.

More recently, artificial intelligence and deep learning approaches have been explored for periapical lesion detection on conventional intraoral radiographs, reporting high diagnostic performance primarily for radiographically evident lesions [13,14]. However, most available studies focus on visually apparent pathology and rely on convolutional neural networks functioning as black-box models, offering limited interpretability. In contrast, radiomics provides explicit and reproducible texture descriptors that may reveal early or visually occult pathological changes while maintaining methodological transparency. Recent studies demonstrate that artificial intelligence and deep learning algorithms can enhance the visualization and identification of subtle periapical changes on panoramic radiographs [15]. Improved visualization supports the more accurate diagnosis of pulp-related periapical disease and facilitates timely therapeutic intervention

The aim of this study was to develop and evaluate a radiomics-based, texture analysis approach for estimating the probability of periapical lesions on conventional intraoral periapical radiographs that are not detectable via visual inspection but are confirmed on cone-beam computed tomography.

## 2. Materials and Methods

### 2.1. Overview of Analysis Pipeline

This study’s workflow consisted of (1) the selection of matched RVG–CBCT image pairs, (2) ROI definition and labeling based on the CBCT findings, (3) radiomic feature extraction from RVG images, and (4) supervised classification using logistic regression. Model training and testing were performed using nested cross-validation with grouping at the examination level to prevent information leakage. All analyses were conducted at the ROI level unless stated otherwise.

### 2.2. Study Design

This retrospective, single-center observational study was conducted in accordance with the Declaration of Helsinki and relevant institutional guidelines. Ethical approval was obtained from the institutional review board and the Local Bioethics Committee (approval number 106/KBL/OIL/2024, 12 December 2024). Owing to the retrospective nature of this study and the exclusive use of fully anonymized radiographic data, the requirement for informed consent was waived.

Matched intraoral periapical radiographs (RVG) and cone-beam computed tomography (CBCT) examination results were retrospectively collected from patients treated at the University Dental Clinic, Department of Endodontics, Kraków, Poland, during routine diagnostic procedures. Prescreening and image selection were performed by experienced clinicians as part of standard clinical care. The final dataset comprised 56 RVG-CBCT image pairs representing various tooth types, including incisors, premolars, and molars. All radiographic data were fully anonymized prior to analysis in accordance with institutional bioethics approval and contained no patient-identifiable information.

All included cases involved adult patients of both sexes aged between 25 and 61 years, with fully developed permanent dentition. Eligible cases were selected based on strict radiographic and clinical criteria to ensure diagnostic consistency and minimize confounding factors. Adult patients with fully developed permanent dentition and closed root apices were included, provided that radiographs demonstrated adequate diagnostic quality with appropriate exposure, contrast, and the absence of motion artifacts. Teeth were required to show no clinical or radiographic signs of trauma, resorption, or large restorations that might obscure the periapical area. Each case included a matched RVG–CBCT image pair, with the same anatomical region captured in both modalities. In multirooted teeth, analysis was limited to roots with clearly visible apices on RVG; when more than one root met this criterion, the buccal root was preferentially selected due to its more consistent visibility and lower risk of anatomical superimposition compared to palatal roots. Patients with systemic conditions known to affect bone metabolism or lesion detectability—such as uncontrolled diabetes, osteoporosis, chronic corticosteroid use, or a history of radiotherapy to the head and neck—were excluded. Additional exclusion criteria included periapical regions overlapping with anatomical structures such as the maxillary sinus or mental foramen, which could compromise both visual and radiomic assessments. Endodontically treated teeth exhibiting extruded root filling materials or intraradicular posts were also excluded, as these artifacts can influence grayscale intensity patterns and texture analysis. All included cases underwent a two-step screening process conducted by experienced clinicians to ensure compliance with these selection criteria before inclusion in the radiomic analysis.

As a result of the deidentification process, only the information confirming adult patient status was retained, while data on sex and precise age were not available. Consequently, the analyses were not stratified by sex or narrow age categories, which is appropriate given the methodological focus of the present study rather than being an epidemiological investigation.

### 2.3. Image Acquisition and Viewing Conditions

All images analyzed in this study consisted of intraoral periapical radiographs acquired between 2022 and 2024 during routine dental diagnostic procedures using standard digital radiography equipment. The radiographs were archived in DICOM format and assessed at their native spatial resolution without any form of image compression. Images were interpreted using high-resolution, medical-grade diagnostic monitors (Bracco Imaging S.p.A., Milan, Italy) with 4K ultra-high-definition capability and a native resolution of 3840 × 2160 pixels.

All radiographic images were reviewed in a dedicated radiology reading room using calibrated, medical-grade 4K monitors (DICOM Part 14-compliant). Ambient lighting was maintained between 25 and 40 lux to reduce glare and visual fatigue. Standard tools such as magnification and contrast adjustment were permitted. Observers were given time to adapt to the lighting environment prior to assessment.

### 2.4. Radiographic Imaging Protocol

Intraoral periapical radiographs were acquired using a digital dental imaging system (Gendex 765 DC paired with the RVG GXS-700 sensor, Gendex Dental System, Des Plaines, IL, USA). Standard imaging parameters included a tube voltage of 65 kVp, a tube current of 7 mA, and an average exposure duration of 0.125 s. The resulting images were captured at a resolution of 1200 × 1600 pixels with a pixel spacing of 0.018 mm. All digital radiographs were saved in 16-bit DICOM format and managed using the institutional PACS (Picture Archiving and Communication System).

Cone-beam computed tomography (CBCT) scans were obtained using an Orthopantomograph KaVo OP 3D Pro (Instrumentarium Dental, PaloDEx Group Oy, Tuusula, Finland), which supports cephalometric imaging. Scans were performed with the patient in a seated position, utilizing standard bite blocks and head stabilizers. Depending on clinical requirements, either a 5 × 5 cm or 8 × 15 cm field of view was chosen. Images were reconstructed from acquisitions with a 125 μm isotropic voxel size, captured during a full 360° gantry rotation. Exposure settings were a 90 kV tube voltage and a 6–8 mA current, with a scan time of approximately 10 s.

### 2.5. ROI Definition and Radiomic Feature Extraction

This single-center study used matched intraoral periapical radiographs (RVG) and cone-beam CT (CBCT) examinations. For each of the 56 RVG images, a corresponding CBCT scan of the same region and patient was available (56 RVG-CBCT pairs).

Eligible RVG-CBCT pairs were identified retrospectively. A diagnostically challenging cohort was assembled by selecting cases in which CBCT demonstrated a periapical lesion while no corresponding periapical radiolucency was visually apparent on the RVG in the same region. These sites were labeled onlyCBCT (CBCT-positive, RVG-negative; lesions occult on RVG). In addition, pairs in which CBCT confirmed the absence of a periapical lesion and RVG showed no periapical radiolucency were included as noLesion (true-negative sites).

Accordingly, 109 regions of interest (ROIs) were grouped into two diagnostic categories:

- noLesion (*n* = 45): No periapical lesion on CBCT and no visible periapical radiolucency on RVG.
- onlyCBCT (*n* = 64): Periapical lesion present on CBCT but without detectable periapical radiolucency on RVG.

**Figure 1:**
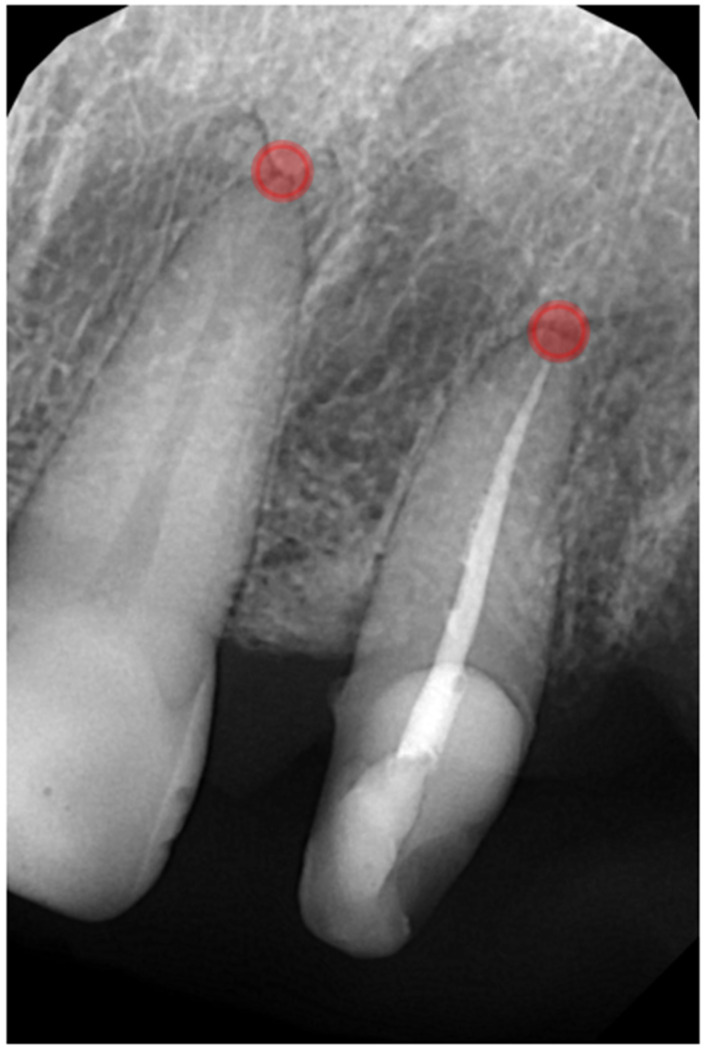
Example periapical radiograph with overlaid circular regions of interest used for radiomic analysis. All ROIs are displayed with a fixed radius of 40 pixels, corresponding to the sampling area used for radiomic feature extraction.

All annotations were performed on the RVG images using the VGG Image Annotator (version 2.0.12). For each tooth/ROI, a single point was placed in the periapical region at the radiographically visible root apex on RVG (Figure 1). The apex location was cross-checked on the matching CBCT to ensure anatomical correspondence between modalities. Point coordinates (pixel space) and the categorical label (noLesion vs. onlyCBCT) were stored and used for subsequent ROI extraction and analysis.

Each point served as the center of a circular ROI. The main analysis used a radius of 40 pixels, which was chosen to cover the anatomical region in which periapical pathology would be expected, while simultaneously limiting overlap with adjacent root apices and avoiding excessive extension beyond the image boundaries.

For each radius, we documented the number of features with valid *p*-values, the number of features with *p* < 0.05 and FDR-adjusted *p* < 0.05, and logistic regression performance. These quantities were plotted against ROI radius to summarize how enlarging the ROI affected the number of statistically significant radiomic features and the discriminative performance of the classifier.

Each ROI was processed with PyRadiomics (version 3.0.1) [10]. The final feature set per ROI consisted of texture descriptors from the following:

- Gray-level co-occurrence matrix (GLCM).
- Gray-level dependence matrix (GLDM).
- Gray-level run length matrix (GLRLM).
- Gray-level size zone matrix (GLSZM).
- Neighboring gray tone difference matrix (NGTDM).

### 2.6. ROI Radius

To assess the influence of ROI radius on radiomic feature significance and classifier performance, the entire workflow was repeated using circular ROIs of radii of 20, 30, 40, 50, 60, and 70 pixels. Feature counts, significance metrics, and classification outcomes were documented for each radius.

### 2.7. Statistical Analysis

All analyses were performed at the ROI level and restricted to the onlyCBCT and noLesion groups. For each feature and radius, the onlyCBCT and noLesion distributions were compared using a two-sided Mann–Whitney U test. Effect size was quantified using Cliff’s delta, a nonparametric measure of stochastic dominance ranging from −1 to +1, where 0 indicates no difference between groups, and larger magnitude indicates stronger separation. The absolute value of Cliff’s delta was used to rank features by effect size; standard thresholds were used to classify negligible (|δ| < 0.147), small (0.147 ≤ |δ| < 0.33), medium (0.33 ≤ |δ| < 0.474), and large (|δ| ≥ 0.474) [16]. To control for multiple testing across all features, *p*-values were adjusted using the Benjamini–Hochberg procedure. Features with a false discovery rate (FDR)-adjusted *p*-value < 0.05 were considered statistically significant.

### 2.8. Machine Learning Pipeline

Using radiomic features extracted from circular ROIs with a radius of 40 pixels, a logistic regression classifier was trained to distinguish ROIs corresponding to CBCT-confirmed periapical lesions that were visually occult on RVG (onlyCBCT) from truly negative sites (noLesion).

The classification task was designed to distinguish ROIs corresponding to CBCT-confirmed periapical lesions that were visually occult on intraoral periapical radiographs (“onlyCBCT”) from ROIs representing truly negative sites (“noLesion”), using radiomic information extracted from RVG images. The unit of analysis was the ROI. For each ROI, radiomic feature extraction resulted in a fixed-length quantitative descriptor of the masked ROI patch, and these descriptors were aggregated into a machine learning dataset. Accordingly, the model input consisted of ROI-level radiomic feature vectors (numerical texture-related features derived from RVG), whereas the model output was a binary class assignment (“onlyCBCT” vs. “noLesion”).

Because multiple ROIs could originate from the same paired RVG–CBCT examination, all data partitioning was performed with grouping at the examination level (RVG–CBCT pair) to prevent information leakage between training and test sets. This ensured that all ROIs from a given examination were assigned exclusively to either the training or testing partition within a given split.

A supervised learning pipeline was implemented in scikit-learn (v1.8.0) and comprised the z-score standardization of the radiomic feature vectors (StandardScaler; mean-centering and unit variance scaling) followed by logistic regression (LBFGS solver). Performance was estimated using nested cross-validation with five outer folds and three inner folds while preserving examination-level grouping in both loops. Within each outer iteration, model settings were selected using only the outer training data in the inner loop, after which the model was refitted on the full outer training partition and applied to the held-out outer test fold. For each outer test fold, the ROC AUC (Receiver Operating Characteristic Area Under the Curve), accuracy, F1-score, and sensitivity and specificity derived from the confusion matrix were recorded and summarized across folds.

### 2.9. Visual Assessment and Metric Computation

For the subset of CBCT-confirmed periapical lesions that were radiographically occult on intraoral periapical radiographs (onlyCBCT, *n* = 64), visual assessment was performed independently by three radiologists. Each reader evaluated the predefined periapical region and assigned a confidence score using a 5-point Likert scale (1 = definitely no lesion; 2 = probably no lesion; 3 = indeterminate; 4 = probably lesion present; 5 = definitely lesion present). Readers were blinded to the CBCT findings and the results of the radiomic analysis.

CBCT served as the reference standard, and all cases in this subset were considered true positives (CBCT label = 1). For quantitative analysis, a predefined positivity threshold of Likert scores 4–5 was used to indicate the visual detection of a lesion. Reader sensitivity was calculated as the proportion of CBCT-confirmed lesions assigned scores of 4 or 5, and the false negative rate was defined as one minus sensitivity. The distribution of Likert scores was additionally summarized to quantify the proportions of lesions rated as absent (scores 1–2), indeterminate (score 3), or detected (scores 4–5).

For comparison, the radiomic model produced a continuous probability score representing the likelihood of a periapical lesion. Model sensitivity was computed, corresponding to the proportion of CBCT-confirmed lesions correctly identified by the model. All metrics were calculated at the case level and reported as percentages. This analysis specifically targeted lesions that were visually occult on conventional radiographs, thereby isolating the diagnostic challenge addressed by the proposed radiomics-based approach.

## 3. Results

### 3.1. Radiomic Feature Statistics

For the standard ROI radius of 40 pixels, 75 radiomic features had valid values in both groups and were included in the univariate analysis. Among these, 44 features showed a raw Mann–Whitney *p*-value < 0.05, and all of them remained statistically significant after Benjamini–Hochberg correction. All FDR-significant features exhibited small or medium effect sizes according to Cliff’s delta; most were classified as medium (32/44), with the remaining classified as small (12/44).

**Figure 2:**
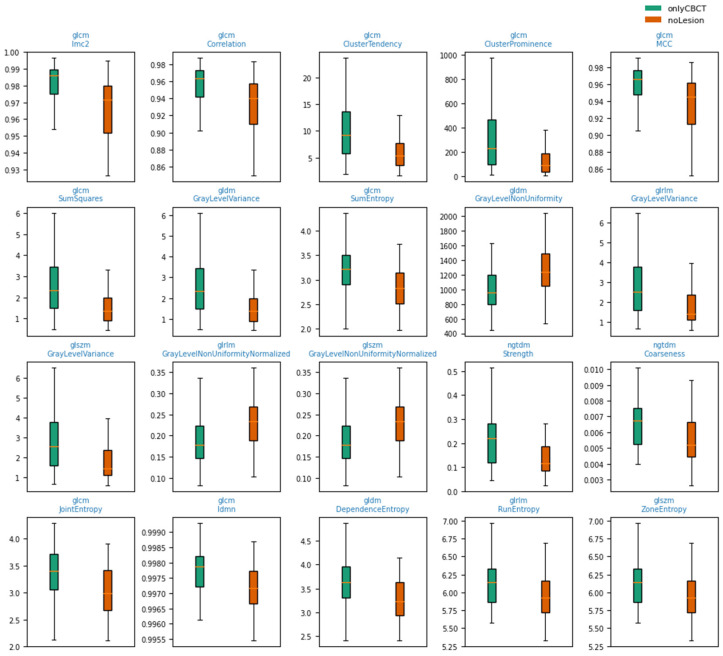
Boxplots of the 20 radiomic texture features with the largest absolute Cliff’s delta among those remaining significant after FDR correction (p_fdr < 0.05), comparing ROIs with CBCT-only lesions (onlyCBCT, green) to truly negative sites (noLesion, orange).

The 20 features with the largest absolute Cliff’s delta among the FDR-significant set were dominated by gray-level texture measures from the GLCM, GLDM, GLRLM, GLSZM, and NGTDM. Several second-order GLCM features showed higher median values in the onlyCBCT group compared to noLesion, including Imc2, Cluster Prominence, Cluster Tendency, Sum Squares, Correlation, MCC, Sum Entropy, Idmn, and Joint Entropy. In contrast, gray-level non-uniformity metrics (GLRLM/GLSZM GrayLevelNonUniformity and their normalized counterparts) tended to be lower in onlyCBCT, while variance-related measures (GrayLevelVariance from GLDM/GLRLM/GLSZM) were higher in onlyCBCT. Taken together, these patterns indicate systematic, medium-magnitude differences in gray-level texture between CBCT-only lesion ROIs and truly negative sites on RVG. The distribution of the 20 radiomic texture features with the largest absolute Cliff’s delta is depicted in Figure 2. In each outer cross-validation fold, approximately 80% of RVG–CBCT pairs were used for model training and hyperparameter tuning (inner loop), while the remaining 20% were held out for independent testing. This procedure was repeated across five outer folds so that each examination contributed to the test set exactly once.

### 3.2. Classification Performance

**Figure 3:**
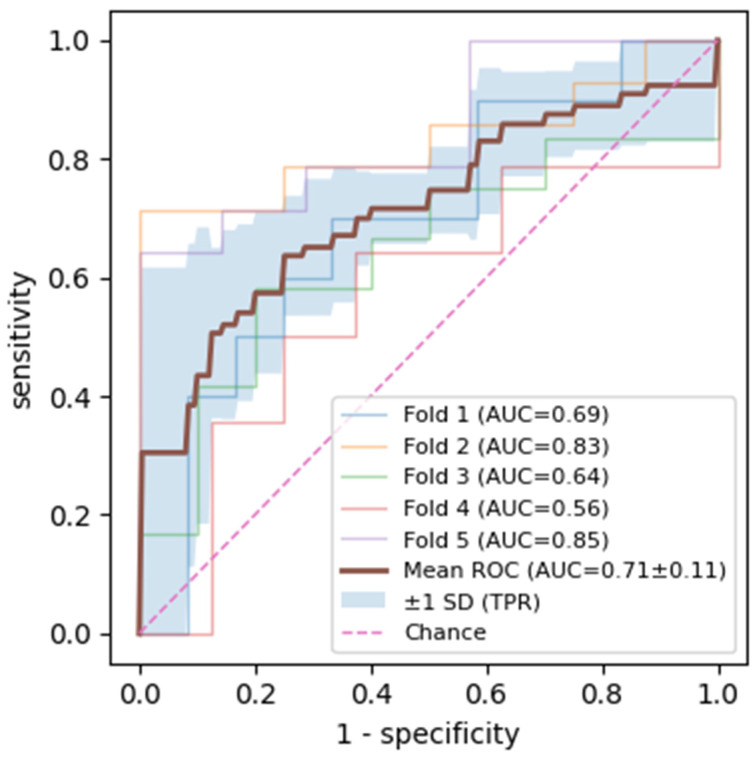
The receiver operating characteristic (ROC) curves of the logistic regression classifier obtained from five-fold grouped cross-validation. Colored curves correspond to individual folds with their respective AUC values, the thick brown line represents the mean ROC curve (mean AUC = 0.71 ± 0.11), and the shaded gray area denotes the ±1 standard deviation band around the mean.

Using the radiomic features extracted with a radius of 40 pixels, a logistic regression classifier was trained to distinguish onlyCBCT from noLesion ROIs. Performance was evaluated using nested grouped cross-validation with five outer folds and three inner folds for hyperparameter tuning, in which grouping was performed at the imaging examination (RVG-CBCT pair) level to prevent information leakage between training and test folds. Across the five outer test folds, the AUC ranged from 0.563 to 0.847, with a mean AUC of 0.715 ± 0.122 (Figure 3). The mean accuracy was 0.680 ± 0.082, with a mean sensitivity of 0.730 ± 0.053 and mean specificity of 0.605 ± 0.230.

### 3.3. Influence of ROI Radius

**Figure 4:**
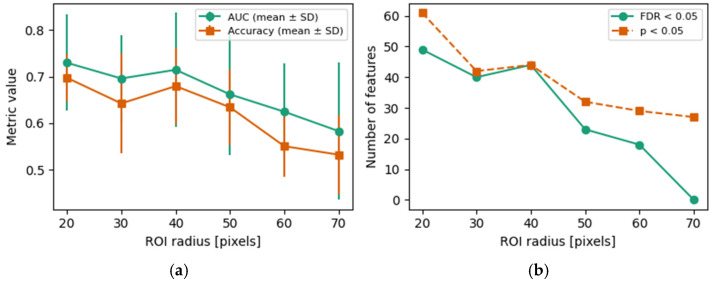
Influence of ROI radius on radiomic classification performance and feature significance. (**a**) Mean ROC AUC and accuracy (±1 SD across five-fold cross-validation) of logistic regression classifier as function of ROI radius. (**b**) Number of radiomic features with raw *p* < 0.05 and with FDR-adjusted *p* < 0.05 (Mann–Whitney U test) for each radius.

To examine the influence of ROI size, the complete radiomics and logistic regression workflow was repeated for circular ROIs with radii of 20, 30, 40, 50, 60, and 70 pixels. For all radii, 75 features had valid *p*-values. The number of features with raw significance (*p* < 0.05) decreased monotonically from 61 (r = 20) to 27 (r = 70). Similarly, the number of FDR-significant features (Benjamini–Hochberg, p_fdr < 0.05) decreased from 49 (r = 20) to 40 (r = 30) and 44 (r = 40), further dropping to 23 (r = 50) and 18 (r = 60) and reaching 0 at r = 70 (Figure 4).

Classifier performance followed the same trend. For r = 20–40 pixels, the mean AUC across the five outer folds was 0.730 ± 0.103 (r = 20), 0.696 ± 0.094 (r = 30), and 0.715 ± 0.122 (r = 40), with corresponding mean accuracies of 0.697 ± 0.050, 0.642 ± 0.107, and 0.680 ± 0.082, respectively. Increasing the ROI radius beyond 50 pixels resulted in a consistent deterioration: the AUC decreased to 0.662 ± 0.131 (r = 50), 0.625 ± 0.104 (r = 60), and 0.583 ± 0.148 (r = 70), while accuracy fell to 0.634 ± 0.081, 0.551 ± 0.065, and 0.532 ± 0.085, respectively. Collectively, these results indicate that smaller ROIs (20–40 pixels) maximize both the number of statistically significant radiomic features and discriminative performance, whereas larger ROIs (≥50 pixels) reduce feature-level significance and substantially degrade classification accuracy and the AUC.

### 3.4. Inter-Reader Agreement and Comparison with Radiomics

**Figure 5:**
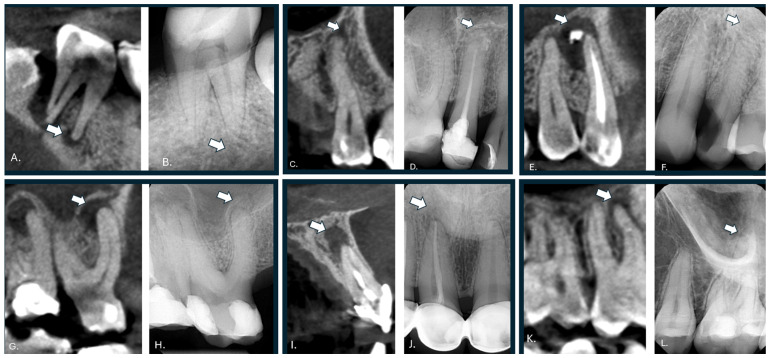
This figure presents tooth CT images (**A**,**C**,**E**,**G**,**I**,**K**) and RVG (**B**,**D**,**F**,**H**,**J**,**L**) in pairs, where arrows indicate lesions for which visualization was uncertain for medical professionals but evident on CT images.

Inter-reader agreement for the visual assessment of CBCT-confirmed periapical lesions that were radiographically occult on RVG was poor. Quadratic-weighted Cohen’s κ values ranged from −0.09 to 0.06 across reader pairs, and overall concordance was negligible (Kendall’s W = 0.015), thus indicating substantial variability in visual confidence scoring. Using a positivity threshold of Likert scores 4–5, reader sensitivities were 20.3%, 15.6%, and 18.8%, corresponding to false negative rates exceeding 80% for all readers. In contrast, the radiomic model achieved a sensitivity of 73.0%, thus markedly outperforming visual assessment by all individual readers. These findings highlight both the limited reliability of human visual interpretation for sub-visual periapical pathology and the potential of radiomics-based decision support to reduce missed CBCT-confirmed lesions. Representative examples of CBCT-confirmed periapical lesions that were visually occult on intraoral radiographs, but evident on CT, are shown in Figure 5.

**Figure 6:**
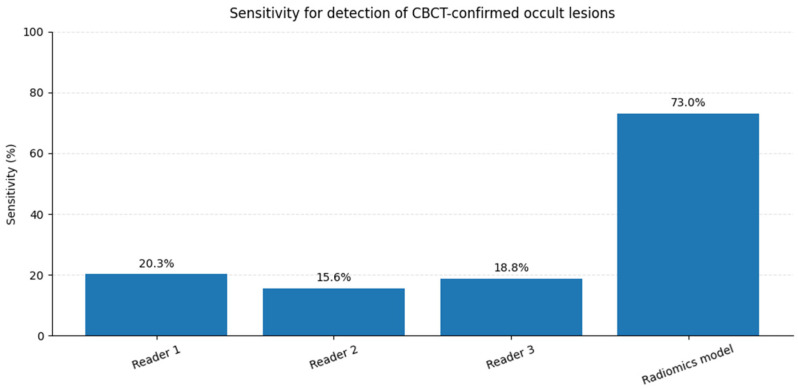
Sensitivity of visual assessment conducted by three radiologists compared with radiomic model for detection of CBCT-confirmed periapical lesions that were radiographically occult on intraoral periapical radiographs (*n* = 64). All lesions were confirmed on cone-beam computed tomography and were not visually detectable on conventional radiographs.

Inter-reader agreement metrics (onlyCBCT, *n* = 64) based on ordinal agreement (5-point Likert scale) are as follows: Quadratic-weighted Cohen’s κ: Reader 1 vs. Reader 2: κ = 0.063; Reader 1 vs. Reader 3: κ = 0.015 < Reader 2 vs. Reader 3: κ = −0.090. Overall concordance (Kendall’s W): W = 0.015. Agreement among radiologists was poor to negligible, thus indicating substantial variability in the visual interpretation of CBCT-confirmed periapical lesions that are occult on RVG. This quantitatively supports the clinical difficulty of detecting sub-visual pathology using conventional radiographs alone. A comparison of reader sensitivity and radiomic model performance is summarized in Figure 6.

## 4. Discussion

Conventional radiographic imaging remains the primary method for detecting periapical lesions, as radiolucencies reflect mineral loss secondary to chronic inflammation originating from the necrotic pulp [17]. However, radiographic changes become visible only after a substantial degree of bone resorption has occurred, which limits early diagnosis. Consequently, advanced image analysis approaches have gained importance for improving the detection and assessment of periapical bone rarefactions associated with pulp pathology.

Cone-beam computed tomography (CBCT) provides additional diagnostic benefit through three-dimensional visualization, enabling the detection of subtle cortical and cancellous bone changes. The pronounced imbalance in detection performance between visual assessment conducted by radiologists and the radiomics-based system should be interpreted in the context of the study design and case selection. All cases included in this analysis were deliberately selected to represent CBCT-confirmed periapical lesions that were not visually detectable on intraoral periapical radiographs, thereby constituting a diagnostically challenging subset of pathology. As cone-beam computed tomography served as the reference standard, the presence of periapical lesions in these cases was independently verified and cannot be attributed to false positive findings. Consequently, the low sensitivity observed for human readers reflects the intrinsic limitation of the visual inspection of conventional radiographs in detecting sub-visual or early periapical changes, rather than reader inexperience or methodological bias. In contrast, the radiomic model was specifically trained to capture subtle texture alterations that are not perceptible to the human eye, which explains its markedly higher sensitivity in this CBCT-positive, RVG-negative cohort.

Comparative studies indicate that CBCT detects periapical lesions in approximately 90–95% of cases, compared with 60–75% for conventional periapical radiography [18]. Although CBCT substantially improves lesion detection relative to two-dimensional imaging, its residual diagnostic limitations support the need for adjunctive analytical methods capable of enhancing lesion recognition on existing radiographic datasets.

Texture-based image analysis methods have demonstrated measurable improvements in the detection and characterization of periapical lesions compared with visual assessment alone. Li et al. [19] showed that convolutional neural networks analyzing texture patterns on periapical radiographs achieved detection accuracies of approximately 85–90%, representing an improvement of nearly 20 percentage points over conventional radiographic interpretation, which typically yields accuracies around 65–70%. Texture analysis applied to CBCT images has also proven effective for lesion characterization. De Rosa et al. [11] reported that gray-level texture features enabled differentiation between periapical granulomas and radicular cysts with classification accuracies of 80–88%, thus substantially exceeding the performance of subjective radiologic assessment. Similarly, Gonçalves et al. [12] demonstrated that CBCT texture analysis improved the detection of furcation-related bone lesions by approximately 15–20% compared with standard visual evaluation, with area-under-the-curve values exceeding 0.80. Collectively, these findings indicate that texture-based approaches can capture sub-visual structural heterogeneity associated with inflammatory bone changes, thus leading to quantifiable gains in diagnostic performance. Beyond improving diagnostic accuracy, such quantitative descriptors may also facilitate risk stratification, the longitudinal monitoring of lesion response to therapy, and integration into machine learning-based decision support systems, thereby reinforcing the role of CBCT radiomics as a valuable adjunct to conventional imaging interpretation.

In the present study, the radiomic analysis of conventional periapical radiographs enabled the discrimination of CBCT-only lesions from truly negative sites, thus achieving a mean AUC of 0.71, a mean accuracy of 68%, and a mean sensitivity of 73%. In contrast to many AI studies that focus on radiographically apparent periapical lesions, this work addresses a clinically more challenging and relevant scenario—lesions that are confirmed on CBCT but visually occult on conventional periapical radiographs. While recent deep learning approaches have reported very high performance for lesion detection on conventional radiographs, with AUC values up to 0.98 and sensitivities of 85–90% [20] or accuracies exceeding 95% following CNN-based image enhancement [21], these results predominantly reflect the detection of overt radiolucencies. Meta-analyses and narrative reviews consistently indicate that AI can improve diagnostic accuracy by approximately 15–30 percentage points compared with unaided human interpretation, again primarily for visually recognizable lesions [22,23].

Earlier deep learning studies reported high diagnostic performance for periapical lesion detection—with accuracies ranging from 82% to 92% and AUCs reaching up to 0.95—our study focused on a diagnostically more challenging subset: CBCT-confirmed lesions that were radiographically occult on RVG. For instance, Ekert et al. [13] reported an AUC of 0.85 and sensitivity of 0.65 for apical lesion detection using CNNs on panoramic images, and the AUC increased to 0.95 when diagnostic agreement was broadened. Similarly, Pauwels et al. [14] observed CNN accuracies between 82 and 90%, but their models were primarily trained on radiographically visible lesions. Other studies, such as those by Endres et al. and Ver Berne et al. [24,25], also reported strong performances (AUCs > 0.90), although they relied on datasets in which lesions were evident to human observers or confirmed via histopathology. These models, while impressive, generally do not address the diagnostic difficulty posed by sub-visual pathology.

In contrast to the above, our method demonstrated consistent diagnostic performance on visually occult lesions, albeit with lower values compared to CNNs trained on radiographically evident pathology. Although these values are lower than those reported in conventional CNN-based models, they reflect the inherently higher complexity of our task and highlight the potential of radiomics as a decision support tool in early or uncertain lesion detection scenarios. Importantly, radiomics offers interpretable texture descriptors rather than black-box predictions, which enhances clinical transparency and could complement deep learning models in future hybrid systems. Our findings support the view that even visually occult periapical lesions can exhibit quantifiable radiomic patterns and suggest that radiomics could aid clinicians in identifying early-stage pathology that would otherwise go unnoticed on conventional imaging.

This moderate performance reflects the diagnostic difficulty of sub-visual lesion detection, which our radiomics-based model was specifically designed to address. Nevertheless, these results demonstrate that clinically meaningful radiomic texture signatures of periapical disease are present even when lesions are not discernible to the human observer. Accordingly, radiomics may be viewed as a complementary, clinically oriented decision support approach rather than a direct competitor to deep learning systems optimized for the benchmark detection of visually obvious lesions. In this context, the classification task was binary, distinguishing regions of interest corresponding to CBCT-confirmed but radiographically occult periapical lesions from truly negative sites with no lesion on either modality.

The diagnostic performance observed in this study should therefore be interpreted within the context of existing artificial intelligence- and radiomics-based approaches for periapical lesion detection on conventional radiographs. Although most published studies report higher performance metrics, these differences largely reflect variations in study design, lesion visibility, reference standards, and analytical objectives.

Beyond endodontics, recent evidence highlights the expanding role of artificial intelligence in dental imaging, particularly in implantology. A systematic review by Macrì et al. [26] documented the growing application of AI for radiographic analysis, bone assessment, and decision support in dental implant planning. Neji et al. [27] provided a clinically oriented synthesis of AI-powered predictive models across implant workflows—including digital planning, risk assessment, peri-implant disease monitoring, and outcome prediction. Although focused on a different clinical application, these developments conceptually align with the present study by underscoring the value of quantitative image analysis for extracting clinically relevant information from conventional radiographs. More broadly, recent reviews on radiomics in clinical radiology emphasize the transformative potential of multimodal approaches that fuse image-based radiomic features with text-derived (e.g., radiology reports via NLP) and clinical data streams. Such integration leverages complementary strengths—subtle imaging phenotypes from radiomics alongside contextual semantic knowledge from reports and patient variables—to enhance model robustness, interpretability, and performance in real-world settings, paving the way for precision decision support systems in dentistry and beyond [28].

An important methodological consideration in this study was the influence of ROI radius on both radiomic feature significance and classification performance. Our results demonstrated that smaller ROIs (20–40 pixels) consistently yielded higher AUC values, greater classification accuracy, and a larger number of statistically significant radiomic features compared to larger ROIs. In contrast, increasing the radius beyond 50 pixels resulted in a marked decline in model performance and reduced feature-level discriminability. This suggests that overly large sampling regions may dilute local texture information by incorporating irrelevant anatomical structures or overlapping adjacent roots. Therefore, the careful calibration of ROI size is essential to optimize the sensitivity of radiomic analysis in periapical imaging. These findings underscore the need for anatomically and task-specific ROI selection, particularly in radiographs in which subtle grayscale variations carry diagnostic weight.

### 4.1. Study Strengths and Limitations

The main strength of this study lies in its focus on a clinically relevant yet underexplored diagnostic scenario, namely the detection of CBCT-confirmed periapical lesions that are not visually detectable on conventional periapical radiographs. Unlike many previous AI studies that primarily address radiographically apparent lesions, this work targets occult pathology that represents a real diagnostic challenge in daily practice. Additional strengths include the use of paired RVG-CBCT data, thus allowing CBCT to serve as a reliable reference standard, and a radiomics-based methodology that provides interpretable texture features rather than relying on black-box deep learning alone. The systematic evaluation of ROI size effects and the use of nested cross-validation further enhance the methodological rigor and robustness of the findings.

This study also has several limitations. The single-center, retrospective design and the relatively small sample size may limit generalizability and increase susceptibility to selection bias. The exclusion of lesions visible on both RVG and CBCT from quantitative analysis restricted the scope to a binary classification problem and prevented direct comparison across the full spectrum of lesion visibility. In addition, differences in RVG and CBCT acquisition parameters, together with the conversion of both image modalities to 8-bit grayscale intensity ranges (0–255), may have led to the loss of subtle grayscale information that could be relevant for texture-based analysis. Moreover, the achieved diagnostic performance was moderate compared with deep learning benchmarks, thus reflecting both the intrinsic difficulty of detecting radiographically occult lesions and the use of a relatively simple classification model. Finally, the absence of external validation and multicenter data limits conclusions regarding clinical deployment, thereby underscoring the need for larger, prospective studies to confirm and extend these findings.

### 4.2. Focus on Moderate Performance

While the performance of the radiomics-based model in this study was moderate—achieving an AUC of 0.71, an accuracy of 68%, and a sensitivity of 73%—these values must be interpreted considering the high diagnostic difficulty posed by the selected cohort. All analyzed cases were CBCT-confirmed lesions that were visually undetectable on intraoral radiographs, representing a clinically realistic but challenging subset that is often missed during routine evaluation. Unlike most AI studies that report high diagnostic accuracy by training on radiographically obvious lesions, our approach specifically targeted sub-visual pathology, in which visual cues are absent. As such, the model’s performance, though not exceeding that of deep learning benchmarks, demonstrates that quantifiable radiomic differences exist even in occult lesions and that they can be leveraged for meaningful diagnostic support. The moderate metrics reflect both the granularity of the task and the absence of overt radiolucencies, not model inadequacy. This underscores radiomics’ potential to serve as a complementary tool, particularly in early detection scenarios where conventional radiology falls short.

### 4.3. Focus on Limited Sample Size of 8-Bit RVG

Despite the limited sample size and the use of 8-bit RVG images, a substantial subset of texture features demonstrated statistically significant between-group differences with non-negligible Cliff’s delta, and the logistic regression classifier achieved moderate discriminatory performance between CBCT-only lesions and truly negative sites. This indicates that radiomic signatures associated with periapical pathology are present in RVG data even when no abnormality is appreciable on visual inspection. Taken together, these findings provide a strong rationale for acquiring a larger, preferably multicenter cohort to validate the observed effects and to further develop and refine the radiomics-based detection of early or CBCT-only periapical lesions.

## 5. Conclusions

This study demonstrated that radiomic texture analysis applied to intraoral periapical radiographs can detect CBCT-confirmed periapical lesions that are visually occult, achieving moderate diagnostic performance. The classifier reached a mean AUC of 0.71, an accuracy of 68%, and a sensitivity of 73%, thus highlighting its potential as a complementary tool for identifying subtle periapical pathology not visible on conventional imaging.

## Data Availability

All data supporting the findings of this study are available from the corresponding author upon reasonable request.

## Abbreviations

The following abbreviations are used in this manuscript:

AI	Artificial Intelligence
CBCT	Cone-Beam Computed Tomography
FDR	False Discovery Rate
GLCM	Gray-Level Co-Occurrence Matrix
GLDM	Gray-Level Dependence Matrix
GLRLM	Gray-Level Run Length Matrix
GLSZM	Gray-Level Size Zone Matrix
ROI	Region Of Interest
ROC AUC	Receiver Operating Characteristic Area Under the Curve
RVG	Radiovisiography
